# Effect of AKT1 (p. E17K) Hotspot Mutation on Malignant Tumorigenesis and Prognosis

**DOI:** 10.3389/fcell.2020.573599

**Published:** 2020-10-06

**Authors:** Ying Chen, Lan Huang, Yongjian Dong, Changli Tao, Rongxin Zhang, Hongwei Shao, Han Shen

**Affiliations:** Guangdong Province Key Laboratory for Biotechnology Drug Candidates, School of Life Sciences and Biopharmaceutics, Guangdong Pharmaceutical University, Guangzhou, China

**Keywords:** AKT1, E17K, mutation, cancer, prognisis

## Abstract

The substitution of the seventeenth amino acid glutamate by lysine in the homologous structural domain of the Akt1 gene pleckstrin is a somatic cellular mutation found in breast, colorectal, and ovarian cancers, named p. Glu17Lys or E17K. In recent years, a growing number of studies have suggested that this mutation may play a unique role in the development of tumors. In this review article, we describe how AKT1(E17K) mutations stimulate downstream signals that cause cells to emerge transformed; we explore the differential regulation and function of E17K in different physiological and pathological settings; and we also describe the phenomenon that E17K impedes tumor growth by interfering with growth-promoting and chemotherapy-resistant AKT1^low^QCC generation, an intriguing finding that mutants may prolong tumor patient survival by activating feedback mechanisms and disrupting transcription. This review is intended to provide a better understanding of the role of AKT1(E17K) in cancer and to inform the development of AKT1(E17K)-based antitumor strategies.

## Introduction

In many cancer lines, the phosphatidylinositol 3-kinase/protein kinase B (PI3K-AKT/PKB) signaling pathway may be more easily activated by genomic aberrations than other signaling pathways (Brugge et al., 2007). The activation of this signaling pathway triggers the signaling cascade of cancer cell proliferation, survival, invasion, and metabolism (Engelman, 2009).

AKT becomes a core factor in the PI3K-AKT signaling pathway by controlling intracellular levels of phosphatidylinositol-3 kinases (PI3Ks) and has multiple targets that are fully activated downstream of PI3K via membrane translocation and phosphorylation (Hennessy et al., 2005). The Akt1 (v-AKT murine thymoma viral oncogene homolog 1) kinase in the AKT family is the active center of this pathway. Akt1 kinase mutations are not uncommon in tumorigenesis, where glutamate substitution for lysine at the seventeenth amino acid position in its pleckstrin homology (PH) domain has received attention (p. Glu17Lys; referred to as E17K) (Carpten et al., 2007).

Mutations in the PH structural domain of AKT1(E17K) increase the binding of Akt1 to the phosphatidylinositol-3,4,5-trisphosphate (PIP3) ligand, forming new hydrogen bonds that accelerate the transfer of AKT from the cytoplasm to the cell membrane, where it is further phosphorylated (Brugge et al., 2007; Carpten et al., 2007). Fully activated AKT re-localizes to the cytoplasm, nucleus or other intracellular sites, phosphorylates a large number of substrate proteins, and thus regulates cell function (Salhia et al., 2012). In addition, the mutation enhances cell migration in luminal breast cancer cells and resistance to chemotherapy drugs (Salhia et al., 2012; Beaver et al., 2013). However, it is also noteworthy that AKT1(E17K) mutant oncoproteins can also selectively destroy rare, quiescent, chemotherapy-resistant, and tumor-promoting AKT1^low^ quiescent cancer cells (QCC) (Yeh and Ramaswamy, 2015; Alves et al., 2018). Multiple studies have reported significantly longer survival time after treatment in patients carrying the E17K mutation (Alves et al., 2018), suggesting that AKT1(E17K) expression plays a crucial role in its oncogenic/anti-tumor mechanism (Omarini et al., 2018).

Given the complexity of the PI3K-AKT pathway in regulating cancer cell behavior, We highlight recent advances in the mechanism by which AKT1(E17K), a key mutant signal, activates the PI3K pathway, selectively induces specific downstream effects, and is involved in tumor cell growth and protein synthesis, and further reveal that altered AKT1(E17K) signaling may play a key role in regulating malignant tumor growth to better understand the role of E17K hotspot mutations that will help identify new targets for therapeutic intervention.

## PI3K-AKT/PKB Signaling Pathway and AKT1(E17K) Mutation

The PI3K-AKT signaling cascade pathway is complex in regulating the behavioral aspects of cancer cells, and its dysregulation not only leads to cellular malignant transformation, but is also associated with tumor cell migration, adhesion, tumor angiogenesis, and degradation of the extracellular matrix (Hanahan and Weinberg, 2000; Yoeli-Lerner et al., 2005), and is a major target for anticancer drug development (Garcia-Echeverria and Sellers, 2008; Engelman, 2009).

Among the most widely observed mechanisms of PI3K-AKT activation is that upon activation of receptor tyrosine kinases (RTKs) by growth factor (GF), PI3K is recruited to this receptor and specifically phosphorylates the 3rd hydroxyl group on the phosphatidylinositol (PI) ring to produce second messenger inositol analogs such as PIP3, which is responsible for regulating various signal effectors and recruiting them to various activation sites within the plasma membrane, and by binding to the N-terminal PH structural domain of AKT prompting AKT to form plasma membrane targets that activate downstream pathways that regulate basic processes such as cell growth, survival, and metabolism (Vivanco and Sawyers, 2002; Vasudevan and Garraway, 2010).

**Figure 1:**
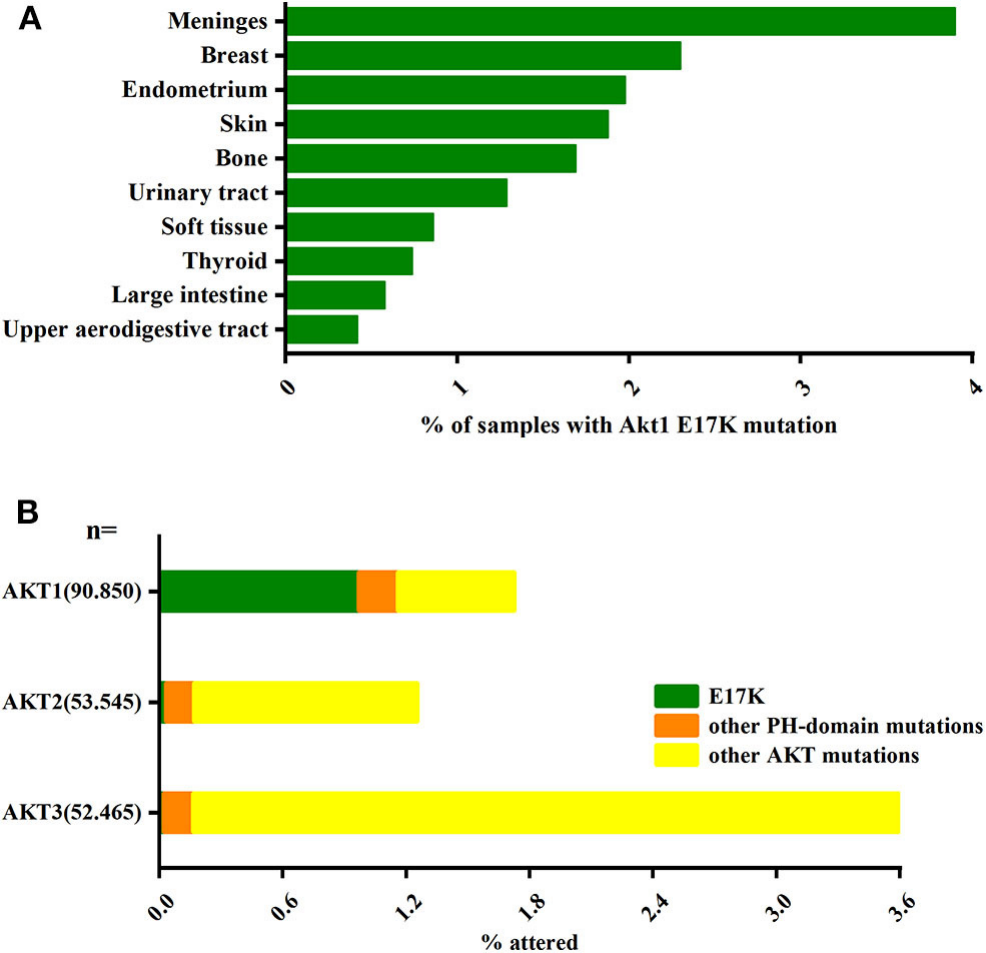
The AKT1(E17K) hotspot is the most characteristic mutation in AKT1. **(A)** Analysis of the distribution of AKT1(E17K) mutations in tumor tissues by COSMIC^1^ database; data Represents the percentage of mutated tissues in each tissue in the tumor sample. **(B)** The frequency of PH domain mutations E17K (green), other PH domain mutations (orange) or other regional mutations (yellow) in the three Akt families (Akt1, Akt2, Akt3) in human cancer patient specimens was analyzed by COSMIC database. The data represent the percentage of the corresponding subtype in all samples. “Activating Akt1 mutations alter DNA double strand break repair and radiosensitivity” by S. Oeck, used under CCBY/adapted from original.

Another major pathway that activates PI3K-AKT is somatic cell mutations in specific components of this signaling pathway. In the AKT gene family, Akt1 plays an important role in cell survival, proliferation, and metabolic activity. And AKT1(E17K) is the gene with the highest frequency of Akt1 mutations and is almost exclusively present in Akt1 (Oeck et al., 2017) (Figure 1). The AKT1(E17K) mutation is a recurrent somatic cell mutation that occurs predominantly in breast cancer [1.8–8% (Adams et al., 1998; Bleeker et al., 2008; Kim et al., 2008; Guo et al., 2010; Lauring et al., 2010; Flatley et al., 2013; Rudolph et al., 2016; Alves et al., 2018)], meningioma [6% (Yesilöz et al., 2017)], ovarian cancer [0.3–8% (Carpten et al., 2007; Eom et al., 2009; de Bruin et al., 2017)], and Proteus syndrome [90% (Lindhurst et al., 2011)] disease, and the mutation is dominated by activation of the PI3K-AKT signaling pathway.

The PI3K-AKT pathway in cancer has been shown to inhibit apoptosis (Zilberman et al., 2009). In previous studies, it has been proposed that AKT1(E17K) mutations induce proprioceptive activation by localizing to the stromal membrane in a PI3K-independent manner (Brugge et al., 2007; Do et al., 2008), promote regeneration and activation of the cell membrane (Furukawa et al., 2005; Shi and Hruban, 2012; Garcia-Carracedo et al., 2013), and stimulate PI3K-AKT signaling, resulting in uncontrolled cell proliferation, increased cell survival, and accelerated cellular invasion (Keniry and Parsons, 2008; Guo et al., 2010; Lauring et al., 2010; Mancini et al., 2016; De Marco et al., 2017). It was shown that the E17K mutation not only promotes membrane localization, but that in the absence of serum the mutant cells exhibit enhanced phosphorylation of serine 473 (Ser473) and threonine 308 (Thr308) [a conformational change after AKT-forming plasma membrane targeting that exposes two key amino acids (Vivanco and Sawyers, 2002; Landgraf et al., 2008)], resulting in constitutive activation of Akt1 (Askham et al., 2010).

Most of the literature demonstrates that AKT1(E17K) mutations or tension protein homolog (PTEN) mutations/deletions are mutually exclusive with PIK3CA mutations (Saal et al., 2005; Abubaker et al., 2007; Carpten et al., 2007; Stemke-Hale et al., 2008; Boormans et al., 2010; Salhia et al., 2012), and show higher levels of phosphorylation of AKT, mTOR, and p70S6 kinases *in vivo* (Stemke-Hale et al., 2008), suggesting that activation of the PI3K pathway by mutations in Akt1 or PIK3CA alone can function as equivalent cells (Lauring et al., 2010).

## The Effects of AKT E17K Mutation Across Various Tumors

Breast cancer (BC) is the malignant neoplasm with the highest prevalence in women (Omarini et al., 2018). An extremely high frequency of AKT1(E17K) mutations has been reported in benign papillary tumors of the mammary gland [up to 20–62% (Dunlap et al., 2010; Troxell et al., 2010; Alves et al., 2018; Mishima et al., 2018)] and is mainly found in BCs with positive estrogen receptor (ER) expression (Stemke-Hale et al., 2008; Lauring et al., 2010). The E17K mutation appears to be present only in lobular and ductal tissue-type breast tumors (Bleeker et al., 2008; Troxell et al., 2010), and recent data show a slightly higher rate of liver and lymph node metastasis in patients with E17K mutation (Smyth et al., 2020b). There are no naturally occurring cell lines with the AKT1(E17K) mutation in human breast cancer cell lines identified to date (Lauring et al., 2010; Beaver et al., 2013). Interestingly, breast cancer patients with the E17K mutation had a higher overall survival rate compared to Akt1 wild type (Salhia et al., 2012; Alves et al., 2018; Smyth et al., 2020b). In addition, recent studies in both *in situ* and aggressive breast cancer have shown that E17K mutations occur more frequently in atypical ductal hyperplasia in early stage ductal carcinoma *in situ* (DCIS) than in aggressive carcinoma (Bleeker et al., 2008; Boormans et al., 2010; Dunlap et al., 2010; Troxell et al., 2010; Kalinsky et al., 2011).

Proteus syndrome (PS) is a rare disease characterized by overgrowth of bone, skin, central nervous system, and other connective tissue (Biesecker, 2006). Of the 29 PS patients tested, 26 (90%) carried the AKT1(E17K) mutation (Lindhurst et al., 2011). The cause of the disease may be related to the occurrence of somatic cell mutations in the embryo, usually manifesting asymmetrical plaque or mosaic-like phenotypes and irregular overgrowth, and a variety of complications may arise during disease development, including skeletal malformations, benign and malignant tumors and some skin damage (Nguyen et al., 2004; Biesecker, 2006; Lindhurst et al., 2014). A study analyzing the phenotype of mice with Akt1 loss of function includes defects in bone remodeling and calcification of chondrocytes in the formation of osteochondralia (Fukai et al., 2010), exhibiting developmental dwarfism and dwarfism in mice. Whereas activated Akt1 can stimulate cartilage calcification *in vitro*, patients with hyperactivated Akt1 have a phenotype of overgrowth and tumor susceptibility, which is the main manifestation of deformity syndrome (Biesecker, 2006; Fukai et al., 2010; Lindhurst et al., 2014). Brain-shaped connective tissue nevus (CCTN) is a highly specific and common lesion in PS patients (Nguyen et al., 2004), and a team of researchers recently identified AKT1(E17K) mutant cells in dermal fibroblasts of CCTN (Lindhurst et al., 2014), suggesting that somatic cell-activating mutations in Akt1 are key determinants of CCTN formation.

In prostate cancer, the mutation appears to have positive clinicopathological features, but is not associated with prostate cancer-specific ductal growth patterns (Boormans et al., 2010). After the early Boormans team first reported a 74-year-old patient with a long cancer-specific survival for ductal adenocarcinoma of the prostate (Boormans et al., 2008), the team evaluated 184 clinical prostate cancer specimens for this mutation and reported an AKT1(E17K) prevalence of 1.4% (Boormans et al., 2010). The team also surprised two prostate cancer patients with AKT1(E17K) mutations and carrying multiple adverse clinical pathologies who were still alive 17 years after diagnosis, suggesting that Akt1 mutations may be associated with good prognosis and survival (Boormans et al., 2008, 2010).

The Akt1 mutation is rare in lung cancer with a total reported frequency of 0.6–5.5%, and studies to date have found that the E17K mutation is mainly expressed in the lung epithelium and that the mutation may be present only in squamous cell carcinoma in non-small cell lung cancer (Bleeker et al., 2008; Do et al., 2008; Kim et al., 2008; Malanga et al., 2008; Rekhtman et al., 2012; De Marco et al., 2015; De Marchi et al., 2019). The AKT1(E17K) mutation promotes migration and invasion of human lung epithelial cells, enhancing their oncogenic and metastatic potential (De Marco et al., 2015). The formed tumors are able to spread in mice and colonize the lungs (Do et al., 2010; De Marco et al., 2017).

## Molecular Mechanism of E17K Mutation Carcinogenesis

**Figure 2:**
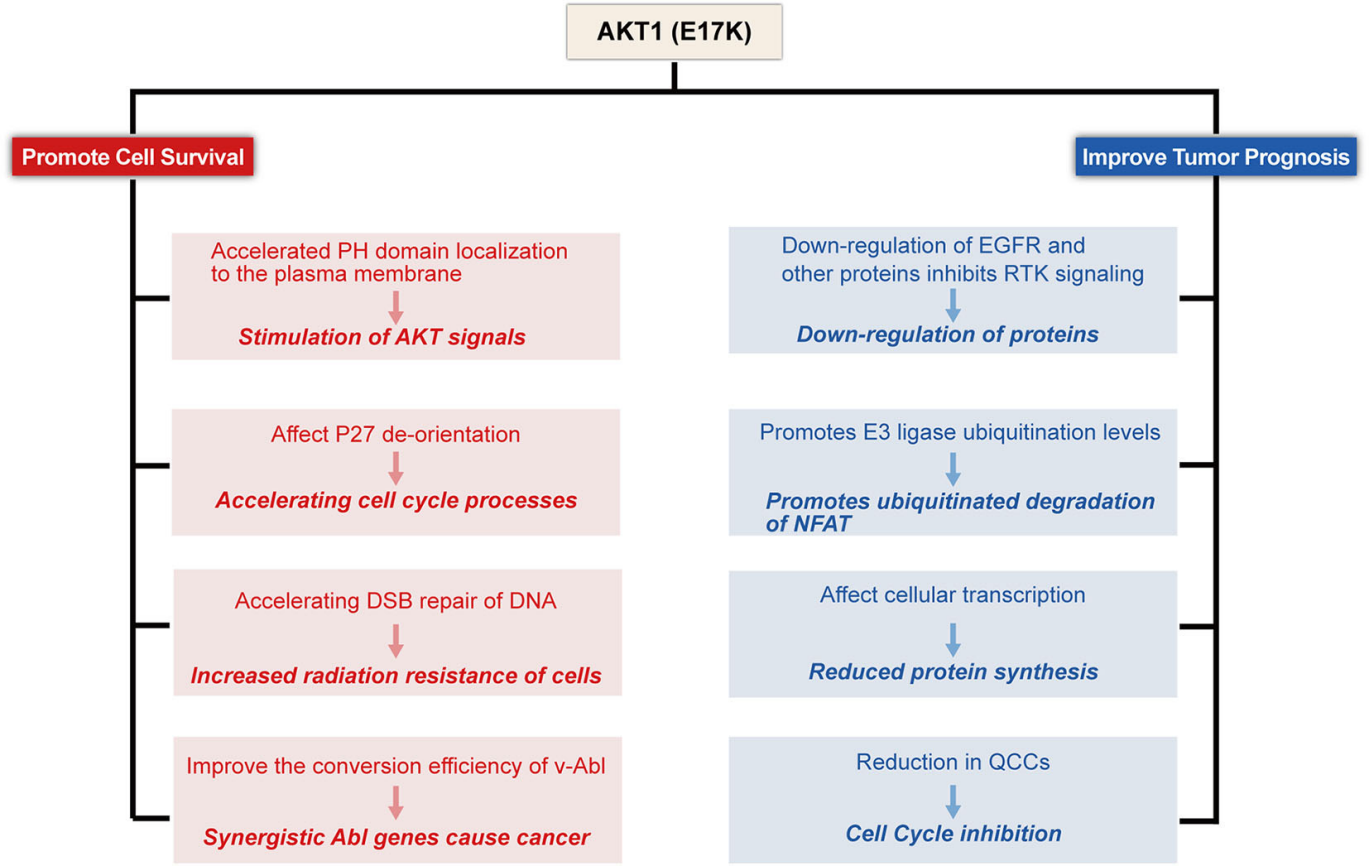
Proposed model for E17K function in Tumor cells. AKT1(E17K) mutations have been shown to have a dual role: oncogenic (depicted in red) and antitumor (depicted in blue). The E17K mutation stimulates downstream signals that cause tumor cells to appear transformatively active; the mutation accelerates cell cycle progression and affects cellular apoptosis; the mutation increases cellular sensitivity to radiation; and synergizes with Abl oncogenes to cause cancer. However, patients with E17K mutated tumors have a favorable prognosis, and we speculate that the E17K mutation inhibits AKT via negative feedback Signaling pathway; Triggering the ubiquitination-proteasome degradation pathway against NFAT by promoting E3 ligase ubiquitination levels; mutations reduce drug-resistant QCCs Cell Content.

**Figure 3:**
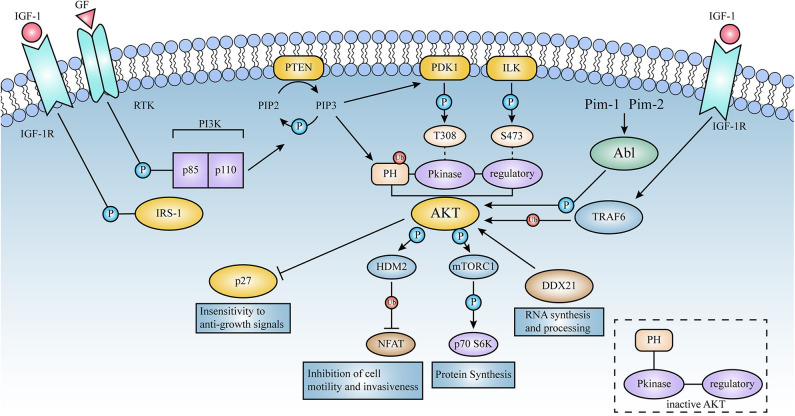
Schematic representation of the signaling pathways activated by AKT. Lines represent either direct or indirect activation (arrow head) or inactivation (blunt end) by AKT. Activation of cell surface growth factor receptors by ligands such as epidermal growth factor and platelet-derived growth factor leads to the automatic phosphorylation of specific tyrosine residues in some of the receptor cells through their intrinsic receptor tyrosine kinase (RTK) activity. In this pathway, PI3K phosphorylates phosphatidylinositol (3,4) diphosphate (PIP2) to phosphatidylinositol (3,4,5) triphosphate (PIP3). This is a key signaling intermediate for PI3K signaling.PIP 3 then induces AKT to form a plasma membrane target through its binding to the N-terminal PH domain of AKT, which changes the kinase from an inactive to an active state. AKT is then phosphorylated on Thr-308 and Ser-473 by PDK1 as well as ILK, respectively. Activated AKT mediates its multiple cellular functions by phosphorylating specific substrates (Figure 2). PTEN is a lipid phosphatase that limits AKT activation by dephosphorylating PIP3 into PIP2.AKT induces a phosphorylation-dependent nucleus-cytoplasmic shuttle of p27, impairing its antiproliferative activity. Compared to AKT 1 (WT), the E17K mutant significantly increased v-Abl translational efficiency and promoted tumorigenesis; these events will be described in detail below.

**Figure 4:**
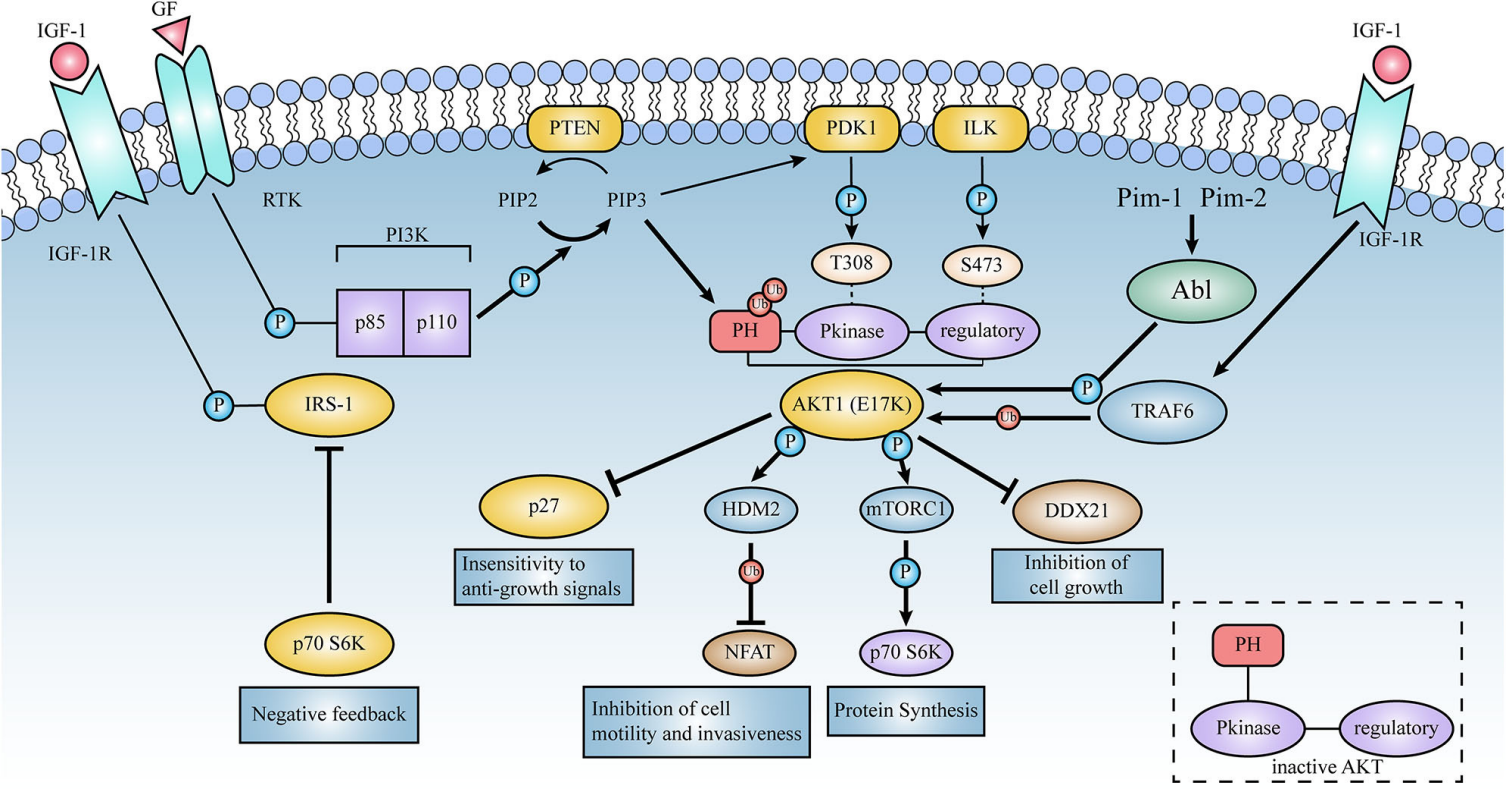
Schematic representation of the signaling pathways activated by AKT1(E17K). Thick lines represent the enhanced effect of AKT1(E17K) mutations on the pathway in contrast to AKT1(WT). E17K-mediated cellular down-regulation of EGFR protein synthesis leads to downstream activation of mTOR and p70S6 kinase in the AKT signaling pathway, which in turn phosphorylates and down-regulates insulin receptor substrate-1 (IRS-1), thereby affecting ligand binding to insulin and insulin-like growth factor 1 (IGF-1) receptors, negatively feedback-regulating AKT activation, and ultimately protein synthesis rate. E17K mutants may mediate disruption of cellular post-transcriptional mechanisms by reducing DDX21 levels. TRAF6 is an E3 ligase that contributes to the localization and phosphorylation of AKT membranes through ubiquitination with the K-63 linkage of AKT. High ubiquitination of E17K promotes phosphorylation of the E3 ligase HDM2, which then targets the NFAT transcription factor for ubiquitin-proteasome degradation, leading to the termination of transcriptional activity; these events are described in detail below.

To date, the molecular and functional role of AKT1(E17K) mutations in human cancer is unknown. Recent studies in xenograft mouse models of melanoma, lung, breast, and colon cancer cells with AKT1(E17K) mutations have shown that AKT1(E17K) has oncogenic activity, manifested by increased proliferation and soft agar colony formation *in vitro* and stimulation of tumor xenograft growth *in vivo* (Boormans et al., 2008, 2010; Stemke-Hale et al., 2008; Zilberman et al., 2009; Lauring et al., 2010; Chandarlapaty et al., 2011; Salhia et al., 2012; Beaver et al., 2013; Davies et al., 2015). Two separate previous studies have shown that activation of AKT in this pathway is not sufficient by itself to induce tumorigenesis, but can be synergistic with other oncogenic signaling cascades, adjunctive to other oncogenic factors-induced tumorigenesis and play a key role in tumor progression (Downward, 1998; Datta et al., 1999; Askham et al., 2010; Lauring et al., 2010; De Marco et al., 2015). This reinforces the finding. Consistent with most of the original observations, AKT1(E17K) mutations regulate cell proliferation through multiple mechanisms (Figures 2–4).

### Stimulation of the PI3K-AKT Signal Pathway

AKT1(E17K) mutations mediate the PI3K-AKT signaling pathway primarily by expanding PIP lipid specificity, causing conformational changes, and enhancing subcellular localization to accelerate the localization of PH structural domain to the plasma membrane, leading to constitutive activation. In addition, mutations amplify the phosphorylation of proteins, as well as enhance kinase activity (Carpten et al., 2007; Malanga et al., 2008; Kumar and Purohit, 2013).

Mutants expand PIP lipid specificity. AKT contains PH structural domain that can bind specifically to PIP3 and are capable of membrane shift and subsequent activation by upstream kinases (Guertin and Sabatini, 2007; Bozulic and Hemmings, 2009). Recent *in vitro* and live cell studies have shown that the main physiological effect of E17K mutations is to expand PIP lipid specificity at the binding site (Falke, 2007; Landgraf et al., 2008; Mancini et al., 2016). This mutation increases the affinity of phosphatidylinositol (4, 5)-bisphosphate (PIP2) by 100-fold and that of PIP3 by 7-fold (Falke, 2007), resulting in enhanced AKT activity and activation of PI3K-AKT signaling (Landgraf et al., 2008; Mancini et al., 2016). Kinetic studies have further revealed that the effect of E17K on PIP lipid binding is mainly due to the electrostatic modulation of the dissociation rate exerted by E17K substitution (Landgraf et al., 2008; Mancini et al., 2016).

Mutants cause rapid conformational changes in the Akt1 PH structural domain. It has been proposed that mutations cause significant alterations in the structural domain of Akt1 PH, with a 4.5-fold increase in its membrane localization, leading to excessive phosphorylation (Carpten et al., 2007; Kim et al., 2008; Shoji et al., 2009). Molecular dynamics simulation studies provide evidence of the observed rapid conformational drift, with a significant increase in the formation of active residues NH bonds involved in protein membrane localization in mutant structures compared to natural structures, resulting in a 4-fold increase in protein membrane localization potential and elevated kinase activity, leading to cancer (Kumar and Purohit, 2013).

E17K mutation results in enhanced subcellular localization. Full activation of mutant E17K requires translocation to the plasma membrane (Yu et al., 2015). It has been shown that the E17K mutation of Akt1 alters the subcellular localization of the protein and increases the transient expression of Akt1 in NIH 3T3 cells. Under serum starvation conditions, AKT1 wild type [AKT1(WT)] was found in the cytoplasm and nucleus, but rapidly transferred to the plasma membrane under platelet-derived growth factor (PDGF) stimulation. In contrast, AKT1(E17K) localizes the protein to the plasma membrane in the absence of an upstream signaling molecule activation state and maintains its own constitutive activation (Carpten et al., 2007). Similar to this study phenomenon, observation of the cellular distribution of the E17K mutant protein by immunofluorescence staining revealed that in the absence of the Abl kinase inhibitor imatinib, a large amount of AKT1(WT) was detected at the edge of the plasma membrane, whereas AKT1(E17K) was mainly detected in the plasma membrane. However, AKT1(WT) was found throughout the cells after treatment with imatinib. In contrast, a large amount of AKT1(E17K) remains localized on the cell membrane (Klejman et al., 2002; Guo et al., 2010). Illustrates that the E17K mutation promotes AKT PH structural domain recruitment to the plasma membrane.

### Oncogenic Factor P27KIP1 (P27) De-localization

The cyclin-dependent kinase inhibitor p27 is a member of the cyclin-dependent protein kinase inhibitory factor (CKI), and many studies have shown that p27 inhibits cell division and proliferation, promotes cell differentiation and apoptosis, and has prognostic value (Pagano et al., 1995; Katayose et al., 1997; Woltman et al., 2003; Zhang et al., 2005). Gain-of-function mutations in AKT1(E17K) trigger abnormal signaling through the PI3K-AKT pathway, affecting p27 de-localization and promoting cell proliferation, migration and invasion to exert its oncogenic activity (De Marco et al., 2015). It has been reported that activated AKT phosphorylates p27 at residues Thr157 and Thr198, induces a phosphorylation-dependent nuclear-cytoplasmic shuttle (out-of-cell nuclear translocation to the cytoplasm) of p27, induces its cytoplasmic redistribution, and impairs its ability to inhibit cell cycle processes (Liang et al., 2002; Shin et al., 2002; Viglietto et al., 2002; Motti et al., 2004), thereby impairing its antiproliferative activity (De Marco et al., 2015).

### Mutants Protect Cancer Cells From Radiation-Induced DNA Damage

Oeck et al. (2017) used a genetic approach to investigate the effects of Akt1 gene alterations on cellular radiation response and found that AKT1(E17K) mutations accelerated the repair of DNA double-strand breaks [DSB (Uematsu et al., 2007; Fraser et al., 2011)], shortened the time for cells to repair radioactively damaged DNA, and improved the survival of embryonic fibroblasts from Akt1 knockout mice after irradiation. The use of AKT inhibitory drugs restored radiosensitivity to AKT1(E17K) expression in tumor cells. In conclusion, AKT1(E17K)-mediated radiation resistance protects cancer cells from radiation-induced DNA damage and can be inhibited by drugs.

### Anti-apoptotic Effect in Collaboration With Abl Oncogene

The Abl oncogene encodes an activated non-receptor tyrosine kinase, which causes cancer in mice and humans. Previous results have shown that v-Abl (a retroviral transduction product of Abl) induced AKT activation is a key signaling pathway in v-Abl-induced cytokine non-dependent cell growth (Oki et al., 2002). Previous studies have found that Pim-1 and Pim-2 kinases are required for v-Abl oncogenic transformation (Chen et al., 2008), and mutant AKT1(E17K) slows the v-Abl inactivation process by reducing the loss of Pim-1 and Pim-2 protein levels (Guo et al., 2010). Furthermore, reports from other groups showed that the E17K mutant is involved in v-Abl-mediated tumorigenesis by significantly increasing the efficiency of v-Abl conversion and enhancing the resistance of the v-Abl transformer to imatinib (an Abl kinase inhibitor) induced apoptosis compared to AKT1(WT).

## Possible Molecular Mechanisms by Which the E17K Mutation Improves Tumor Prognosis

In the current report, although AKT1(E17K) mutations promote leukemia and cell proliferation and survival in some solid tumor cell lines, there is some controversy regarding the relationship between the prognosis of tumor patients. Interestingly, although activation of AKT increases cell survival and promotes tumor growth, there is *in vitro* evidence that activation of AKT inhibits cancer cell migration and invasion and reduces the incidence of metastatic lesions (Hutchinson et al., 2001, 2004; Arboleda et al., 2003; Yoeli-Lerner et al., 2005; Vasudevan and Garraway, 2010). In addition, strong AKT activation may increase oxidative stress, leaving cells vulnerable to reactive oxygen species (ROS)-induced damage. In contrast, AKT-deficient cells are resistant to ROS-induced senescence (Chen et al., 2005; Nogueira et al., 2008). This is a matter of concern. More importantly, multiple groups have demonstrated that the AKT1(E17K) mutation plays a surprising growth inhibitory role in heterozygous grafts such as breast cancer (Salhia et al., 2012; Alves et al., 2018). These studies suggest that AKT1(E17K) mutations may play a dual role in tumorigenesis, not only by activating signaling pathways that act as prokinetic AKT signaling in physiology and disease, but also by inhibiting invasion and metastasis that act as anti-AKT genes (Figure 2).

### E17K Mutation Ultimately Blocks AKT Upstream of the AKT Signal Cascade, Activating the Feedback Loop Pathway

Recently, it has been reported that there may be a feedback loop behind AKT1(E17K)-mediated cellular activity (Bandarage et al., 2009). The data show that epidermal growth factor receptor (EGFR) is inhibited or reduced in E17K-expressing breast cancer cells (Salhia et al., 2012), reduced protein synthesis blocks insulin stimulation and phosphorylation of p70S6K (Schmelzle and Hall, 2000), and negatively regulates AKT activation, ultimately leading to feedback inhibition of PI3K-AKT activation (Haruta et al., 2000; Shah and Hunter, 2004; O'Reilly et al., 2006; Vasudevan and Garraway, 2010). This may be a feedback mechanism to prevent uncontrolled cellular proliferation (Nogueira et al., 2008). E17K-mediated cellular downregulation of EGFR protein synthesis leads to downstream activation of mTOR and p70S6 kinase (mTOR-dependent key regulator of protein translation), among others, in the AKT signaling pathway, which in turn phosphorylates and downregulates insulin receptor substrate-1 (IRS-1), thereby affecting ligand binding to insulin and insulin-like growth factor I (IGF-I) receptors, interfering with AKT activation (Aguirre et al., 2002; Vasudevan and Garraway, 2010), and ultimately affecting protein synthesis rate (Bandarage et al., 2009; Salhia et al., 2012), which in turn inhibits mTOR pathway. This suggests that patients with E17K may have a natural inhibitory effect on the mTOR pathway, further suggesting that E17K may be associated with a good prognosis (Salhia et al., 2012).

In addition, AKT1(E17K) attenuates RTK signaling through feedback inhibition, favorably blocking the formation of mammary tumors driven by HER 2 (Chandarlapaty et al., 2011; Salhia et al., 2012; Mancini et al., 2016). The data showed that mRNA expression, total protein levels, and phosphorylation of RTK were significantly reduced in human tumor tissues carrying AKT1(E17K) (Mancini et al., 2016; Alves et al., 2018).

### Ubiquitination and Phosphorylation

Recent studies have revealed that ubiquitination plays an important role in regulating AKT function (Suizu et al., 2009; Toker, 2009; Yang et al., 2009), providing potential new avenues for pharmacological intervention. Ubiquitination is one of the important ways of post-translational modification of proteins and is important in major life activities such as cell proliferation, apoptosis, and differentiation (Yang et al., 2009, 2010). Ubiquitin proteins can covalently link to one or more lysine (K) residues on intracellular target proteins catalyzed by a series of enzymatic reactions such as ubiquitin activating enzyme (E1), ubiquitin conjugating enzyme (E2), and ubiquitin ligase (E3). Of these, the ubiquitin chain formed at the K48 position mainly mediates proteasomal degradation, while the K63 ubiquitin chain affects protein activity and localization (Bhoj and Chen, 2009; Yang et al., 2010). AKT K48 chains formed by E3 ligases such as MUL1 (Kim et al., 2020), TTC3 (Suizu et al., 2009) and RNF8 (Zhu et al., 2020) were found to mediate AKT protein degradation and inactivation, whereas ubiquitination of AKT K63 residues mediated by TRAF6 (Yang et al., 2009) or SKP2-SCF (Chan et al., 2012) E3 ligases plays a key regulatory role in the membrane localization and phosphorylation activation of AKT. Interestingly, the AKT1(E17K) mutant exhibits stronger levels of ubiquitination than AKT1(WT), and blocking this ubiquitination process significantly reduces Akt membrane recruitment and phosphorylation, while overexpression of TRAF6 can further increase AKT1(E17K) ubiquitination levels (Yang et al., 2009; Li et al., 2013).

It is also noteworthy that activated Akt may regulate the ubiquitination levels of other multiproteins in the cell by phosphorylating other E3 ligases. Since AKT (E17K) exhibits stronger levels of ubiquitination and phosphorylation activation than AKT1(WT) (Yang et al., 2009; Fan et al., 2013; Li et al., 2013; Hechtman et al., 2015), AKT1(E17K) may have greater regulatory capabilities. Studies (Zhou et al., 2001; Yoeli-Lerner et al., 2005) found that activated AKT-mediated phosphorylation of the E3 ligase HDM2 promotes ubiquitination of NFAT (nuclear factor of activated T cells), which then targets NFAT transcription factors for proteasomal degradation, leading to termination of transcriptional activity. Previous studies have shown that aberrantly activated NFAT promotes tumor formation and infiltrative metastasis (Jauliac et al., 2002; Foldynová-Trantírková et al., 2010), and inactivation of NFAT prevents breast cancer cell invasion (Jauliac et al., 2002). Thus, AKT1(E17K) may trigger a ubiquitination-proteasome degradation pathway against NFAT (Stemke-Hale et al., 2008), exerting a role in resisting invasiveness, attenuating cell motility, and blocking tumor progression, which may contribute to improved prognosis.

### Mechanisms That Disrupt Cellular Post-transcription

Disruption of cellular post-transcriptional mechanisms may result in delayed and inefficient protein synthesis and inhibit cell growth. DDX21 is involved in non-coding RNA binding for ribosome-nucleoprotein complex formation and promotes rRNA transcription, processing and modification (Calo et al., 2015). DDX21 was reported to be significantly down-regulated in cells expressing the E17K mutation. In E17K patient samples, DDX21 expression was reduced in the cytoplasm and slightly increased in the nucleus (Salhia et al., 2012).

### Patients With Mutant Tumors Produce Fewer Slow-Growing, Quiescent Cancer Cells (QCC)

A state of drug-resistant, quiescent cancer cells (AKT1^low^QCC) exists in premalignant breast lesions prior to the onset of aggressive disease. QCCs may be present in invasive lesions such as DCIS and are involved in the development and recurrence of aggressive tumors (Dey-Guha et al., 2011; Alves et al., 2018; Kabraji et al., 2019). QCCs are insensitive to radiotherapy and can evade organismal immune clearance, and upon activation can re-enter the cell cycle and replicate, ultimately leading to disease recurrence, complicating diagnosis and treatment of cancer patients and making it difficult to manage (Yeh and Ramaswamy, 2015). This hypothesis is supported by newly obtained results suggesting that AKT1(E17K) protein promotes early clonal amplification by enhancing kinase activity and effectively inhibits tumor growth by reducing the amount of growth-promoting and chemoresistant QCCs, thus explaining the relatively better clinical prognosis and survival of patients with AKTI (E17K) mutant tumors receiving standard adjuvant therapy. AKT1(E17K) as a useful tool to reduce the amount of QCCs provides new evidence for therapeutic response in research tumors and will require more research to more directly link AKT1^low^ cells to the metastatic cascade response, thus providing a new direction for disease research of great significance.

## Specifically Targeted Drug AZD5363

Capivasertib (AZD5363), a drug that specifically targets the AKT signaling pathway, has been shown (Davies et al., 2015) to inhibit downstream signaling of AKT1(E17K) mutations and the growth of breast cancer outgrowth models at the cellular level, and a phase I clinical trial that included 41 patients with advanced solid malignancies (NCT01353781) (Tamura et al., 2016) reported that AZD5363 inhibited downstream signaling in two patients with AKT1(E17K) mutations, showing promising anticancer activity. In addition, AKT1(E17K) mutation-positive tumors may be sensitive to AZD5363.

In a multi-cohort phase I clinical study (NCT01226316) (Hyman et al., 2017), AZD5363 was first targeted to AKT1(E17K) mutations for the treatment of patients with relapsed, refractory advanced cancer. It was reported that 52 patients with cancer carrying the E17K mutation produced a durable response and tumor regression with an overall efficacy rate of 25% and a disease progression-free survival time (PFS) of 5.5–6.6 months. Recently published results from a phase II clinical trial (Smyth et al., 2020a,b) showed that AZD5363 monotherapy for patients with ER-positive metastatic breast cancer with AKT1(E17K) mutations had an objective remission rate (ORR) of 20% compared to 36% in the combination Fulvestrant (an ER receptor antagonist) (Osborne et al., 2004) treatment group. The results confirmed that chemotherapy and radiotherapy outcomes in patients with AKT1(E17K) cancer were improved as monotherapy and in combination with Fulvestrant (Smyth et al., 2020a). This study provides evidence that the AKT1(E17K) mutation is a reasonable therapeutic target for AZD5363 in a variety of cancers. The current Phase III study in AZD5363 development is conducting multiple basket trials testing AZD5363 alone or in combination with other drugs in multiple tumor types to achieve better PFS, which will provide a new option for patients with AKT1(E17K) relapsed refractory disease. In addition, data have shown that patients with double mutations in the same allele of PIK3CA that result in overactivation of PI3K signaling and increased oncogenicity are more sensitive to treatments targeting PI3K (Vasan et al., 2019). Patients with both AKT1 mutations and PI3K pathway mutations have a prolonged duration of therapy (DOT) to mTOR inhibitors (Smyth et al., 2020b). This explains the E17K mutation independent of the PI3K AKT pathway activation status that may be more beneficial to the drug. Future studies will conduct subgroup analyses based on PIK3CA/AKT/PTEN status to explore the appropriate population for AZD5363 in combination with other drug regimens.

## Conclusion

The complexity of signaling pathways activated in solid tumors, the emergence of therapeutic resistance and the evolving cancer genome these intrinsic resistance are major obstacles to successful cancer treatment. Akt1 is a key mediator of signaling pathways involved in cell survival, proliferation and growth. Excessive activation of the Akt1 protein leads to dysregulation of the PI3K-AKT pathway (Khan and Ansari, 2017). Recent studies have identified a single amino acid p. E17K mutation in the PH structural domain of the Akt1 protein, a hotspot mutation that occurs mainly in breast, colorectal, lung and ovarian cancers, leading to important cellular alterations through synergistic other oncogenic signaling cascade pathways and adjuvant other oncogenic factors. Interestingly, the AKT1(E17K) mutation activates the PI3K-AKT pathway on the one hand to enhance cell migration and oncogenic capacity, while on the other hand E17K expression may be involved in the regression process of solid tumors and may be shown to help stop the growth of human solid tumors, positively affecting the survival and prognosis of cancer patients, adding new complexity to the role of this hotspot mutation in cancer progression. Thus, the discovery of the AKT1(E17K) mutation raises the possibility of treating cancer by targeting the mutant Akt1.

In summary, in this paper, the mechanisms and novel aspects of E17K's oncogenic and antitumor effects are highlighted, while explaining why patients with mutations do not fully benefit from treatment with AKT inhibitors. In the future, a specific gene will be subdivided into many different loci and types of mutations, and the global analysis of genes regulated by different activating members may have the ability to develop the most appropriate targeting drugs for each of them.

## Author Contributions

YC and HShe conceived and designed the project. LH and YD analyzed the data. CT and RZ interpreted the data. YC, HSha, and HShe wrote the paper. All authors contributed to the acquisition, analysis, interpretation of data, manuscript drafting and revising, final approval of the version to be submitted and published, and agreement to be accountable for all aspects of the work in ensuring that questions related to the accuracy or integrity of any part of the work are appropriately investigated and resolved.

## Conflict of Interest

The authors declare that the research was conducted in the absence of any commercial or financial relationships that could be construed as a potential conflict of interest.

## Footnotes

^1^*COSMIC: The Catalog of Somatic Mutations in Cancer*. Available online at: https://cancer.sanger.ac.uk/cosmic.
